# Response to the Letter to the Editor by D. Richardson: Analysis of the Interaction of Dp44mT with Human Serum Albumin and Calf Thymus DNA Using Molecular Docking and Spectroscopic Techniques

**DOI:** 10.3390/ijms17111917

**Published:** 2016-11-16

**Authors:** Zhongjie Xu, Youxun Liu, Sufeng Zhou, Yun Fu, Changzheng Li

**Affiliations:** 1 College of Life Science and Technology, Xinxiang Medical University, Xinxiang 453003, China; xcj029@163.com; 2 Department of Molecular Biology & Biochemistry, Xinxiang Medical University, Xinxiang 453003, China; liuyouxun@126.com (Y.L.); sufengzhou@xxmu.edu.cn (S.Z.); fuyun9801@163.com (Y.F.); 3 Henan Collaborative Innovation Center of Molecular Diagnostics and Laboratory Medicine, Xinxiang 453003, China

**Keywords:** Dp44mT, fluorescence quenching, molecular docking, human serum albumin, cytotoxicity, MTT, spectroscopy, circular dichroism

## Abstract

This response refers to:

Xu, Z.; Liu, Y.; Zhou, S.; Fu, Y.; Li, C. Analysis of the Interaction of Dp44mT with Human Serum Albumin and Calf Thymus DNA Using Molecular Docking and Spectroscopic Techniques. *Int. J. Mol. Sci*. **2016**, *17*, 1042.

Merlot, A.M.; Sahni, S.; Lane, D.J.R.; Richardson, V.; Huang, M.L.H.; Kalinowski, D.S.; Richardson, D.R. Letter to the Editor: Analysis of the Interaction of Dp44mT with Human Serum Albumin and Calf Thymus DNA Using Molecular Docking and Spectroscopic Techniques and. *Int. J. Mol. Sci*. **2016**, *17*, 1916.

## Dear Editors,

Thank you for forwarding the concerns (or comments) from Dr. Richardson [1] regarding our paper “Analysis of the Interaction of Dp44mT with Human Serum Albumin and Calf Thymus DNA Using Molecular Docking and Spectroscopic Techniques” [2], which was recently published in the *International Journal of Molecular Sciences*. We are aware of the fact that Dr. Richardson in a leading researcher in the field and both respect Dr. Richardson and regularly refer to the many publications from his group.

Nonetheless, we would like to respond to the specific concerns raised by Dr. Richardson.

In reading the article by Xu, Z., et al. entitled “Analysis of the Interaction of Dp44mT with Human Serum Albumin and Calf Thymus DNA Using Molecular Docking and Spectroscopic Techniques” [Int. J. Mol. Sci. 2016, 17, 1042], there is evidence that some of the key methods and comparisons utilized in this study are non-optimal and problematic.

We would also like to bring to the attention of readers a number of important flaws and factually incorrect statements in this article.

The paper examines the binding of the anti-cancer agent, di-2-pyridylketone 4,4-dimethyl-3-thiosemicarbazone (Dp44mT), to human serum albumin (HSA) and DNA and states (page 1, Introduction para 1) that “no studies have examined the effects of the interaction between Dp44mT and biological molecules, such as proteins and nucleic acids”. However, two studies from our group have previously comprehensively examined the binding of Dp44mT to HSA [Oncotarget 2015, 6, 10374–10398] and DNA [J. Med. Chem. 2006, 49, 6510–6521]. These previous studies performed very similar experiments, including cytotoxicity studies, circular dichroism (CD), fluorescence and molecular docking studies, and hence, the current paper appears as a duplication and cannot be considered a novel contribution to the literature. Significantly, the authors knew of one of these articles as they directly cite it (REF #13 in their manuscript). Such statements appear misleading and inappropriate and this requires correction.

## Reply:

Dr. Richardson’s concerns regarding the sentence “no studies have examined the effects of the interaction between Dp44mT and biological molecules, such as proteins and nucleic acids” in the introduction section are justified and we do realize that the sentence is equivocal. Unfortunately, this sentence was changed as a consequence of language editing of the original manuscript. We are sorry for our carelessness, but we did not have the intention to mislead readers. Consequently, the sentence should be changed back to the original version: “information related to the effects of the interaction between Dp44mT and biological molecules such as human serum albumin (HSA) or DNA has not yet been fully and systematically studied”.

Secondly Dr. Richardson considers our work a duplication and not a novel contribution to the literature. We did read Dr. Richardson’s papers [3,4]. However, what we addressed in our paper was how interaction strength generally affects drug cytotoxicity. To this end, obtaining the thermodynamic parameters of the interaction from spectral data was required. Furthermore, we were aware of the fact that Dr. Richardson published a related spectral study. However, in that particular study, the thermodynamic parameters of interaction of Dp44mT with HSA were not determined. That was why we wanted to perform more experiments in order to obtain the aforementioned parameters to enrich our knowledge related to Dp44mT. Circular dichroism (CD), fluorescence and molecular docking are well accepted and generally used methods for investigating such interactions. There is no duplication, we obtained data beyond the work we cited [3]. Important contributions from our work were: (1) we used UV-visible spectra, FRET, molecular docking, fluorescence titration and so on to obtain the required and previously unknown thermodynamic parameters; (2) we determined the Dp44mT favorable site on HSA by molecular docking (in the aforementioned reference, this data was absent); (3) the sequence specific-binding of Dp44mT to DNA was not evident from our study.

*Moreover, the following methods, comparisons and interpretations of results may be unreliable and should be taken into consideration when interpreting the results presented:*

- ***Characterization and purity of Dp44mT:*** *The authors synthesized Dp44mT in their own laboratory and present only the mass spectrum to confirm its structural identity. Notably, other methods of structural characterization (e.g., 1H and 13C NMR, etc.) and chemical purity (e.g., elemental analysis) are required to ensure that the Dp44mT was indeed correctly synthesized, and was of the high purity necessary for biological studies. As such, the purity of the compound utilized is unclear.*

## Reply:

We do agree with Dr. Richardson’s suggestion that ^1^H-NMR, ^13^C-NMR, elemental analysis, and HPLC used for structural characterization and purity assessment, ensuring Dp44mT was synthesized with high quality for a biological study. Dp44mT was synthesized by reaction of commercial 2-dipyridylketone (Sigma) with *N*,*N*-dimethyl-3-thiosemicarbazide (Sigma). This is a simple one-step condensation reaction, we finished the reaction, and following specific recrystallization, a high purity of Dp44mT was achieved. Purity assessment was performed and in accordance with your suggestion, the requested information is now included in the correction [5]. ^1^H-NMR, ^13^C-NMR, elemental analysis, and HPLC fully supported that the structure of Dp44mT was correct and high purity was achieved.

- ***The MTT-based proliferation studies:*** *The MTT studies performed in this article may be unreliable due to the fact that HSA (one of their key experimental variables) causes false positive results in MTT assays, producing spurious and inaccurate interpretations [Biotechniques 2007, 43, 178–182]. Alternative techniques should have been used, such as viable cell counts. Hence, the results obtained cannot be considered as reliable.*

## Reply:

The MTT-based method is still widely used to assess a drug’s cytotoxicity or proliferation inhibition [6,7,8]. Given that each method has its own advantages and shortcomings, in both the aforementioned methods, use of equal initial cell numbers is critical. Furthermore, measurement after addition of MTT should be objective, except when the drug reacts or has absorption at the measuring wavelength. Although direct cell counts using trypan blue is an alternative method, the counts achieved from several visual fields might still contain unavoidable errors. Both methods depend on the person performing them and their level of training. For this reason, various labs report various IC50 results for the same drug in the same cell line. In our work, for assessing the effect of HSA on drug cytotoxicity, a cell culture system was used without fetal calf serum (FCS; see experimental section), and consequently, the claim that “Serum albumin leads to false-positive results” is unfounded. HSA was used as a control and Dp44mT with the same concentration HSA was used in an experiment group. We therefore feel that, under the given conditions, the obtained MTT data would be objective. It should be noted that, as mentioned in the experimental section, Dp44mT exposure was kept as short as possible exactly because of the lack of FCS; normally, exposure times from 24, 48, to 72 h are used.

- ***Comparison of HepG2 proliferation studies to previous literature:*** *The effect of HSA on the anti-proliferative activity of Dp44mT was performed using HepG2 cells in this article. Notably, the authors then compare the results obtained in this cell line to previous studies [Oncotarget 2015, 6, 10374–10398] and state (Page 2, last paragraph): “growth inhibition induced by Dp44mT in the presence of equimolar HSA was similar to that without HSA, in contrast to previous findings [REF13: Merlot, A.M.; et al. Potentiating the cellular targeting and anti-tumor activity of Dp44mT via binding to human serum albumin: Two saturable mechanisms of Dp44mT uptake by cells. Oncotarget 2015, 6, 10374–10398].” Significantly, the referenced study examined the effect of HSA and Dp44mT on the proliferation of SK-N-MC cells [Oncotarget 2015, 6, 10374–10398], not HepG2 cells, and thus, cannot be directly compared.*

## Reply:

To address the phenomenon of the effect of HSA on drug cytotoxicity, we qualitatively compared our results with the reference’s findings [3], even though the cell line used was different. It should be mentioned that our results were obtained under the same conditions. To evaluate to what extent our research supported the reference’s findings, we also assayed the cytotoxicity of Dp44mT-Cu under the same conditions, which turned out to be similar to those reported. We did not have the intention to challenge the reference’s findings. To ensure that the result was objective, we repeated the analysis several times. It should be pointed out that, during the review process of the manuscript, one referee judged the Dp44mT-Cu data to be superfluous, but we insisted on inclusion of the Dp44mT-Cu data to support the reference’s finding. Therefore, we only addressed the phenomenon of the effect of HSA on drug cytotoxicity, without claiming a direct comparison. The results from your lab and my lab were at specific conditions.

- ***Circular dichroism method—use of DMSO:*** *DMSO has high far-UV absorbance and should not be used in CD measurements [Nat. Protoc. 2006, 1, 2876–2890]. The implementation of DMSO leads to high tension (HT) voltage values and the saturation of the detector [Nat. Protoc. 2006, 1, 2876–2890], leading to erroneous results. Alternative solvents that do not interfere with CD spectra, including ethanol and acetonitrile, should be used to dissolve the ligand for subsequent addition to the protein.*

## Reply:

We do agree with Dr. Richardson’s point of view: DMSO potentially affects the CD spectrum. In our experiments, DMSO was used at very low concentration, and the CD spectra were corrected for its contribution. Hence, the data obtained was objective. Future related CD experiments may use ethanol or acetonitrile if the sample under investigation is soluble in those solvents.

- ***Circular dichroism interpretation:*** *Notably, upon examination of their experimental CD results, the authors state (Page 9, paragraph 1): “In the presence of different concentrations of Dp44mT, a slight reduction in negative ellipticity was observed, indicating that the α-helix content of the protein was decreased (Figure S2). The contents of secondary structure can be calculated based on experimental data [REF36: Kandagal, P.B.; Ashoka, S.; Seetharamappa, J.; Shaikh, S.M.T.; Jadegoud, Y.; Ijare, O.B. Study of the interaction of an anticancer drug with human and bovine serum albumin: Spectroscopic approach. J. Pharm. Biomed. Anal. 2006, 41, 393–399.], but it was limited due to larger errors.” However, the nature of the large error is not well explained. Unfortunately, the authors rely on simulated data to assess the effect of Dp44mT on the secondary structure of HSA. Critically, the simulated secondary structural data for HSA alone reported by the authors does not reflect the known levels of α-helical or β-sheet content of HSA previously published [Nature 1992, 358, 209–215]. Hence, again, there are serious uncertainties regarding these data.*

## Reply:

The contents of the secondary structure can be determined either via direct calculation (based on equations) or, as another option, through simulation-based on experimental data. Because DMSO was used in our CD experiment, the contribution of DMSO to the CD spectra required correction of the spectra. Due to direct calculation for the α-helix content based on a MRE value at 208 nm, after correction for DMSO, the averaged CD curve at given wavelength (208 nm) may be less accurate. Therefore, simulation of the curve may decrease the error upon direct calculation, and consequently we used the simulation program (K2D2) rather than the analytical equation, which is also widely used in CD spectrum simulation [9,10,11].
